# THE ROLE OF MISSINGNESS IN DAILY DIARY DATA

**DOI:** 10.1093/geroni/igad104.1974

**Published:** 2023-12-21

**Authors:** Mustafa Yildiz, Vicki Winstead, Carolyn Pickering

**Affiliations:** University of Alabama at Birmingham, Birmingham, Alabama, United States; University of Alabama at Birmingham, Birmingham, Alabama, United States; University of Alabama at Birmingham, Birmingham, Alabama, United States

## Abstract

Objections are often raised to using intensive daily diary research methods with family caregivers of persons with dementia. These objections include the daily lack of time, stress associated with dementia caregiving, in particular, and depression and anxiety associated with the demands of the caregiving process. It is argued that these factors may deter caregivers from consistent compliance and lead to bias in the data. The aim of this paper is to describe and predict missingness in daily diaries using the data from three micro-longitudinal studies that include 10,680 days of diaries across all three studies. Key indicators and demographic variables were used to predict missing days, which was quantified from compliance data using a latent variable within the framework of a multiple indicators-multiple causes model. Missingness was random and not predicted by demographics or key outcome variables (e.g. stress, depression, race, ethnicity). Findings suggest despite the time demands and stressors of caregiving, caregivers do complete diaries without systematic patterns of missingness. Researchers should not be deterred from doing intensive daily diary protocols with caregivers experiencing psychopathology who may benefit the most from real-time assessment of symptoms.

